# Analysis of the therapeutic gain in the treatment of human osteosarcoma microcolonies in vitro with 211At-labelled monoclonal antibody.

**DOI:** 10.1038/bjc.1994.196

**Published:** 1994-06

**Authors:** R. H. Larsen, O. S. Bruland, P. Hoff, J. Alstad, E. K. Rofstad

**Affiliations:** Department of Chemistry, University of Oslo, Norway.

## Abstract

Microcolonies were obtained by culturing cells of two human osteosarcoma lines (OHS and KPDX) and one human melanoma line (WIX-c) for either 24 or 72 h. The microcolonies were treated with either alpha-particle radiation emitted by the 211At-labelled monoclonal antibody (MAb) TP-3 or external beam X-rays. Survival of microcolonies was assayed by colony formation. Therapeutic gain factor (TGF) values were calculated for two survival levels, 50% and 20% microcolony regeneration (i.e. at least one cell in 50% or 20% of the colonies survived the treatments). The TGF values were affected by the specific activity of the 211At-MAb conjugate, the antigen expression of the cells and the size and growth pattern of the microcolonies. Treatment with 211At-TP-3 gave TGF values that varied from 1.3 +/- 0.4 to 4.5 +/- 0.7 (mean +/- s.e.). The antigen-rich OHS cell line had on average 1.6 times higher TGF than the antigen-poor KPDX cell line. The TGF increased significantly with colony size for the densely packed colonies of the KPDX cell line but not for the OHS cell line, which had colonies with cells growing in a more scattered pattern. Control experiments with the two non-specific 211At forms, free 211At and 211At-labelled bovine serum albumin, gave TGF values from 0.6 +/- 0.1 to 1.0 +/- 0.3. This study suggests that in vivo evaluation of 211At-MAbs using relevant tumour models is desirable.


 © Macmillan Press Ltd., 1994

### Analysis of the therapeutic gain in the treatment of human osteosarcoma microcolonies *in vitro* with $^{211}$At-labelled monoclonal antibody

R.H. Larsen[1,2], Ø.S. Bruland[3,4], P. Hoff[1], J. Alstad[1] & E.K. Rofstad[2]

[1]*Department of Chemistry, Section D, University of Oslo, Blindern, 0315 Oslo, Norway; Departments of* [2]*Biophysics,* [3]*Tumor Biology and* [4]*Oncology, The Norwegian Radium Hospital, Montebello, 0310 Oslo, Norway.*

**Summary** Microcolonies were obtained by culturing cells of two human osteosarcoma lines (OHS and KPDX) and one human melanoma line (WIX-c) for either 24 or 72 h. The microcolonies were treated with either α-particle radiation emitted by the $^{211}$At-labelled monoclonal antibody (MAb) TP-3 or external beam X-rays. Survival of microcolonies was assayed by colony formation. Therapeutic gain factor (TGF) values were calculated for two survival levels, 50% and 20% microcolony regeneration (i.e. at least one cell in 50% or 20% of the colonies survived the treatments). The TGF values were affected by the specific activity of the $^{211}$At-MAb conjugate, the antigen expression of the cells and the size and growth pattern of the microcolonies. Treatment with $^{211}$At-TP-3 gave TGF values that varied from $1.3 \pm 0.4$ to $4.5 \pm 0.7$ (mean ± s.e.). The antigen-rich OHS cell line had on average 1.6 times higher TGF than the antigen-poor KPDX cell line. The TGF increased significantly with colony size for the densely packed colonies of the KPDX cell line but not for the OHS cell line, which had colonies with cells growing in a more scattered pattern. Control experiments with the two non-specific $^{211}$At forms, free $^{211}$At and $^{211}$At-labelled bovine serum albumin, gave TGF values from $0.6 \pm 0.1$ to $1.0 \pm 0.3$. This study suggests that *in vivo* evaluation of $^{211}$At-MAbs using relevant tumour models is desirable.

Compounds labelled with the α-particle emitter $^{211}$At have for some time been preclinically investigated as a means for irradiation of tumour cells (Bloomer *et al.*, 1981; Brown *et al.*, 1981; Vaughan *et al.*, 1981, 1982; Harrison & Royle, 1987; Link *et al.*, 1989). Although therapeutic effects have been achieved with compartmentally delivered non-specific $^{211}$At preparations in murine models (Bloomer *et al.*, 1984; Vergote *et al.*, 1992), the use of $^{211}$At-labelled compounds with some degree of selective tumour uptake seems to be the most promising strategy (Humm, 1987; Humm & Chin, 1993). The therapeutic potential of α-emitters is increased when they are coupled to molecules with high tumour affinity because of the short range and high ionisation density of α-particles (Brown, 1986; Kozak *et al.*, 1986; Kurtzman *et al.*, 1988; Macklis *et al.*, 1989).

We have recently studied the cytotoxicity of $^{211}$At-TP-3 monoclonal antibody (MAb) on single-cell suspensions of three human osteosarcoma cell lines (OHS, SAOS and KPDX) (Larsen *et al.*, 1994). The study showed that the sensitivity to $^{211}$At-TP-3 treatment was governed by cellular properties other than those governing sensitivity to treatment with external beam X-rays. The cellular property most important for sensitivity to $^{211}$At-TP-3 was the antigen density. The cell inactivation was found to increase substantially with increasing specific activity of the $^{211}$At-TP-3 preparation. At high specific activities, the cytotoxic effect of $^{211}$At-TP-3 was significantly higher than that of non-specific $^{211}$At-labelled bovine serum albumin (BSA). It was concluded that $^{211}$At-TP-3 of high specific activity may have the potential to give clinically favourable therapeutic ratios in the treatment of osteosarcoma.

The clinical conditions for many types of cancer are normally very different from the conditions in the single-cell suspension model. Osteosarcoma, for instance, has a strong tendency to metastasise by cells being trapped in the bone marrow sinusoids and lung capillaries. Cells in microcolonies infiltrating or growing adjacent to normal tissue will have binding kinetic conditions different from free-floating single cells. Steric hindrance from adjacent cells may reduce the number of antigens available for circulating MAbs and, hence, reduce the potential of radioimmunotherapy (RIT). On the other hand, radiation cross-fire from radiolabelled antibody molecules bound to antigens of neighbouring cells may enhance the efficacy of RIT against tumour cell colonies.

In the present paper we have extended our *in vitro* investigation of α-particle RIT from the single-cell suspension model to a surface-deposited microcolony model. A surface-deposited microcolony model was chosen rather than a multicellular spheroid model to avoid the large penetration barrier for MAbs usually seen in spheroids. We used two human osteosarcoma cell lines (OHS and KPDX) with very different antigen expression, and one human malignant melanoma cell line (WIX-c) with no significant antigen expression for the sarcoma-associated MAb TP-3 (Bruland *et al.*, 1986). Microcolonies were grown on a plastic surface in tissue culture flasks. Inactivation was studied following treatment with $^{211}$At-TP-3 MAb, free $^{211}$At, $^{211}$At-BSA and external beam X-rays. The objective of this study was to measure and quantify the therapeutic gain from $^{211}$At-labelled MAb on antigen-positive versus antigen-negative microcolonies and to determine some parameters important for RIT of tumour micrometastases.

### Materials and methods

### *Cell lines*

The two human osteosarcoma cell lines, OHS and KPDX (Bruland *et al.*, 1985; Fodstad *et al.*, 1986), and the human melanoma cell line, WIX-c (Rofstad *et al.*, 1991), have been described in detail previously. WIX-c is more resistant to X-rays than OHS and KPDX. The X-ray survival curve parameters have been found to be: $D_o = 0.60 \pm 0.03$ Gy, $n = 9.9 \pm 2.2$ (OHS); $D_o = 0.82 \pm 0.10$ Gy, $n = 3.7 \pm 1.2$ (KPDX) (Larsen *et al.*, 1994); and $D_o = 0.98 \pm 0.04$ Gy, $n = 2.9 \pm 1.1$ (WIX-c) (Rofstad, 1992).

The cell lines were cultured in monolayer in RPMI-1640 medium (25 mM HEPES and L-glutamine) supplemented with 13% fetal calf serum, 250 mg $l^{-1}$ penicillin and 50 mg $l^{-1}$ streptomycin. The cells were incubated at 37°C in a humidified atmosphere of 5% carbon dioxide in air and subcultured every 5–7 days after treatment with 0.05% trypsin/0.02% EDTA. Cells in passages 50–100 *in vitro* were used in the

Correspondence: R.H. Larsen, Department of Chemistry, Section D, University of Oslo, PO Box 1033 Blindern, 0315 Oslo, Norway.
Received 11 November 1993; and in revised form 20 January 1994.

present work. The cell lines were found to be free from *Mycoplasma* contamination by using the Hoechst fluorescence and the mycotrim methods.

*Monoclonal antibody and antigen expression*

The MAb TP-3 of subclass IgG2b, which recognises an 80 kDa antigen commonly expressed on sarcoma cells (Bruland *et al.*, 1988), was used. Production and purification have been described elsewhere (Bruland *et al.*, 1986). The average numbers of antigens per cell were $7.4 \times 10^5$ and $1.2 \times 10^5$ for OHS and KPDX respectively (Larsen *et al.*, 1994). WIX-c had no significant antigen expression for TP-3.

*Production of $^{211}$At*

$^{211}$At (half-life 7.2 h) was produced by the $^{209}$Bi(α,2n)$^{211}$At reaction at the cyclotron at Oslo University. After separation from the bismuth target (Larsen *et al.*, 1993), the astatine was coupled to the MAb or BSA using the ATE method (Zalutsky & Narula, 1987; Garg *et al.*, 1989). The procedures for astatine labelling of MAbs have been described in detail elsewhere (Zalutsky *et al.*, 1989). The immunoreactivities were measured according to previously published procedures (Lindmo *et al.*, 1984) using OHS cells and were between 55% and 75%. Solutions with free $^{211}$At were made by dissolving elementary astatine in phosphate-buffered saline (pH 7.4).

*Microcolonies*

Cells in exponential growth were harvested by trypsinisation and plated in various numbers in 25 cm$^2$ tissue culture flasks (Nunclon) containing $1 \times 10^5$ lethally irradiated (30 Gy) feeder cells, which were plated 24 h earlier. It was verified experimentally that the feeder cells themselves did not give rise to colonies and that the use of feeder cells increased and stabilised the plating efficiency (PE). The cells were incubated at 37°C in a humidified atmosphere of 5% carbon dioxide in air for 24 or 72 h for formation of microcolonies. The size and growth pattern of the microcolonies were examined by light microscopy before irradiation. Three control flasks and three flasks containing irradiated microcolonies were used to determine each survival level in each experiment.

The number of cells in the microcolonies is presented in Table I. The cells in the 24 h microcolonies were difficult to count exactly because of difficulty in distinguishing viable cells from clonogenically inactive feeder cells. However, a maximum number of cells per colony was established.

*External beam X-ray irradiation*

X-ray irradiation was performed at a dose rate of 4.0 Gy min$^{-1}$ using a Müller RT 250 X-ray unit operated at 250 kV and 20 mA, and with 0.5 mm copper filtration of the beam (Rofstad, 1992).

*Incubation with $^{211}$At preparations*

After addition of $^{211}$At preparations, the flasks were incubated on a tilting platform for 90 min. The platform was tilted carefully to avoid loss of cells from the surface-bound microcolonies. The flasks were then placed in an incubator and the $^{211}$At was allowed to decay completely. Activity levels of 10, 25 and 50 kBq per ml of tissue culture medium (initial activity) were used.

**Table I** Number of cells in microcolonies

| *Cell line* | *24 h microcolonies* | *72 h microcolonies* |
|---|---|---|
| OHS | 1–4 | 13.5 ± 0.7 |
| KPDX | 1–4 | 11.5 ± 0.6 |
| WIX-c | 1–4 | 12.1 ± 1.0 |

The cells were counted using a stereomicroscope. The values are based on data from four individual experiments (72 h microcolony values: mean ± s.e.).

*Clonogenic assay*

The cells were incubated for 1–3 weeks after the radiation exposure. The culture medium was replaced approximately once a week. The colonies were fixed in ethanol, stained with methylene blue and counted using a stereomicroscope. The surviving fraction (SF) of microcolonies was determined as follows:

SF = (number of colonies after treatment)/
(number of colonies in untreated control)

*Curve fitting, therapeutic gain calculations and statistical analysis*

Survival curves were fitted to the data using linear dose–log survival interpolation between experimental points. The doses (medium activities) to give at least one surviving cell in 50% [$D_{50}$ ($A_{50}$)] and 20% [$D_{20}$ $(A)_{20}$)] of the microcolonies were found by linear dose (medium activity)–log survival interpolation between two experimental points. Standard errors (s.e.) were calculated from the s.e. of the two nearest experimental points. Therapeutic gain factor (TGF) for $^{211}$At-TP-3 RIT relative to X-ray treatment was calculated according to the equation (e.g. OHS, 50% colony survival):

$$TGF_{50} = [D_{50}\ (\text{X-rays, OHS})/A_{50}(^{211}\text{At-TP-3, OHS})]/[D_{50}(\text{X-rays, WIX-c})/A_{50}(^{211}\text{At-TP-3, WIX-c})]$$

i.e., the antigen positive osteosarcoma cell lines were used as models for tumour tissue and the antigen-negative melanoma cell line was used as model for normal tissue. TGF for $^{211}$At-TP-3 RIT was also calculated according to the equation (e.g., KPDX, 20% colony survival):

$$TGF_{20} = [A_{20}(^{211}\text{At-BSA, KPDX})]/[A_{20}(^{211}\text{At-TP-3, KPDX})]$$

i.e. the tumour tissue damage was modelled by the cell inactivation caused by the $^{211}$At-TP-3 treatment and the normal tissue damage by the cell inactivation caused by the $^{211}$AT-BSA treatment.

The TGF values were calculated using both 50% and 20% survival of microcolonies. One-way analysis of variance followed by a Student–Newman–Keuls test was used to identify differences between cell lines. A significance level of $P = 0.05$ was used.

### Results

*X-ray irradiation*

The external beam X-ray survival curves for microcolonies are presented in Figure 1. The survival increased with microcolony size, in agreement with the increase in cell number per microcolony and the PE of the single cells in the microcolonies. The WIX-c cell line was more resistant than the OHS and KPDX cell lines ($P < 0.05$), which had a similar response to X-rays. The $D_{50}$ and $D_{20}$ values for X-ray irradiation of microcolonies are presented in Table II.

*$^{211}$AT-TP-3 MAb*

The microcolony survival curves for OHS, KPDX and WIX-c incubated with $^{211}$At-TP-3 are shown in Figure 2. Figure 2a presents the curves for 24 h microcolonies incubated with $^{211}$At-TP-3 of 14 MBq mg$^{-1}$. The curves for 24 h microcolonies exposed to $^{211}$At-TP-3 of 50 MBq mg$^{-1}$ are shown in Figure 2b. A comparison of Figure 2a and 2b shows that the increase in specific activity resulted in reduced regeneration of microcolonies for the OHS line, whereas the KPDX line showed a significant difference only at the 50 kBq ml$^{-1}$ medium activity level. In Figure 2c the survival curves for 72 h microcolonies are presented. At the 10 kBq ml$^{-1}$ activity level the difference between the survival of OHS and KPDX was minor. At higher medium activities the OHS line was affected considerably more than the KPDX line.

The survival for both OHS and KPDX was clearly lower than that for WIX-c at all activity levels. $A_{50}$ and $A_{20}$ values for OHS, KPDX and WIX-c incubated with $^{211}$At-TP-3 MAb are presented in Table III. For the 24 h microcolonies incubated with $^{211}$At-TP-3 of 14 MBq $mg^{-1}$, OHS and KPDX had similar $A_{50}$ values. Otherwise, OHS had significantly

**Figure 1** OHS (○), KPDX (□) and WIX-c (●) microcolonies irradiated with X-rays 24 h (**a**) or 72 h (**b**) after plating of single cells on a plastic surface in tissue culture flasks. Survival fraction points represent geometrical mean (± s.e.) calculated from 3–5 experiments.

**Table II** $D_{50}$ and $D_{20}$ values for external beam X-ray irradiation of microcolonies

| Cell line | *24 h microcolonies* $D_{50}$ (Gy) | $D_{20}$ (Gy) | *72 h microcolonies* $D_{50}$ (Gy) | $D_{20}$ (Gy) |
|---|---|---|---|---|
| OHS | 1.5 ± 0.1 | 3.0 ± 0.4 | 1.6 ± 0.2 | 3.6 ± 0.5 |
| KPDX | 1.5 ± 0.1 | 3.1 ± 0.7 | 2.0 ± 0.1 | 4.3 ± 0.4 |
| WIX-c | 3.3 ± 0.2 | 4.9 ± 0.6 | 4.2 ± 0.5 | 6.0 ± 1.2 |

The dose (± s.e.) in Gy to give 50% ($D_{50}$) and 20% ($D_{20}$) survival of microcolonies calculated using linear dose–log survival interpolation between experimental points (Figure 1). s.e. values were calculated from the s.e. values of the two nearest experimental points.

**Figure 2** OHS (○), KPDX (□) and WIX-c (●) microcolonies exposed to $^{211}$At-TP-3 monoclonal antibody with a specific activity of 14 MBq $mg^{-1}$ 24 h after plating (**a**), 50 MBq $mg^{-1}$ 24 h after plating (**b**) and 50 MBq $mg^{-1}$ 72 h after plating (**c**) of single cells on a plastic surface in tissue culture flasks. The activity was allowed to decay completely. Survival fraction points represent geometrical mean (± s.e.) calculated from 3–6 experiments.

lower $A_{50}$ and $A_{20}$ values than KPDX ($P<0.05$), and both had significantly lower $A_{50}$ and $A_{20}$ values than WIX-c ($P<0.05$). There was no significant change in survival of 24 h microcolonies of WIX-c caused by the increase in specific activity from 14 MBq mg$^{-1}$ to 50 MBq mg$^{-1}$ of the $^{211}$At-MAb.

### *$^{211}$At-BSA and free $^{211}$At*

The microcolony survival curves for $^{211}$At-BSA and free $^{211}$At are presented in Figures 3 and 4. At activity levels of 25 and 50 kBq ml$^{-1}$, free $^{211}$At was significantly more toxic than $^{211}$At-BSA ($P<0.05$). The curves for 24 h microcolonies of WIX-c incubated with $^{211}$At-BSA and $^{211}$At-TP-3 are similar. $A_{50}$ and $A_{20}$ values for the two forms are presented in Table IV.

### *Therapeutic gain factor*

TGF values were calculated for the treatment of OHS and KPDX microcolonies with $^{211}$At-TP-3 MAb by using WIX-c (Table V) or treatment with $^{211}$At-BSA (Table VI) as references. $TGF_{50}$ and $TGF_{20}$ were consistently above unity and were on average a factor of 1.6 larger for OHS than for KPDX, and this agrees with the higher antigen expression of the OHS cell line.

TGF values were also calculated for the non-specific $^{211}$At forms free $^{211}$At and $^{211}$At-BSA for comparison (Table V). There was no significant difference between the two osteosarcoma cell lines. The values were varying from 0.6 ± 0.1 to 1.0 ± 0.3 (mean ± s.e.) and there was no significant difference between free $^{211}$At and $^{211}$At-BSA.

**Table III** $A_{50}$ and $A_{20}$ values for $^{211}$At-TP-3 added to the culture medium of microcolonies

| *Cell line* | *OHS* | *KPDX* | *WIX-c* |
|---|---|---|---|
| 24 h microcolonies | | | |
| 14 MBq mg$^{-1}$ | | | |
| $A_{50}$ | 5.2 ± 0.9 | 5.8 ± 0.7 | 28.4 ± 1.0 |
| $A_{20}$ | 15.7 ± 3.1 | 24.6 ± 0.7 | 49.6 ± 9.1 |
| 50 MBq mg$^{-1}$ | | | |
| $A_{50}$ | 3.3 ± 0.3 | 6.8 ± 0.5 | 25.9 ± 1.0 |
| $A_{20}$ | 7.7 ± 1.1 | 20.1 ± 3.9 | 46.9 ± 6.8 |
| 72 h microcolonies | | | |
| 50 MBq mg$^{-1}$ | | | |
| $A_{50}$ | 6.0 ± 0.7 | 8.0 ± 0.6 | 46.3 ± 3.7 |
| $A_{20}$ | 14.5 ± 1.5 | 28.4 ± 3.1 | [a] |

Added activity (± s.e.) in kBq ml$^{-1}$ in the culture medium to give 50% ($A_{50}$) and 20% ($A_{20}$) survival of microcolonies calculated using linear activity-log survival interpolation between experimental points (Figure 2). The $^{211}$At preparations were allowed to decay completely (> 72 h) before the medium was changed. s.e. values were calculated from the s.e. values of the two nearest experimental points. [a]The 20% survival level was outside the region covered by experimental data for WIX-c.

**Figure 3** OHS (○), KPDX (□) and WIX-c (●) microcolonies exposed to $^{211}$At-BSA 24 h after plating of single cells on plastic surface in tissue culture flasks. The activity was allowed to decay completely. Survival fraction points represent geometrical mean (± s.e.) calculated from six experiments.

### Discussion

Therapeutic gain measured *in vitro* reflects to a large extent the experimental conditions. It is noteworthy that the experimental procedures used here did not allow any strong mixing of the cells and the $^{211}$At-MAb preparations, and that the unbound $^{211}$At preparations were allowed to decay completely in the growth medium, giving a high non-specific dose to the cells. Nevertheless, significant therapeutic gain was achieved with $^{211}$At-TP-3 on antigen-positive cells. The parameter most strongly influencing the therapeutic gain was the specific activity of the $^{211}$At-MAb preparation. Owing to

**Figure 4** OHS (○), KPDX (□) and WIX-c (●) microcolonies exposed to free $^{211}$At 24 h after plating of single cells on plastic surface in tissue culture flasks. The activity was allowed to decay completely. Survival fraction points represent geometrical mean (± s.e.) calculated from six experiments.

**Table IV** $A_{50}$ and $A_{20}$ values for $^{211}$At and $^{211}$At-BSA added to the culture medium of microcolonies

| *Cell line* | *OHS* | *KPDX* | *WIX-c* |
|---|---|---|---|
| 24 h microcolonies | | | |
| Free $^{211}$At | | | |
| $A_{50}$ | 12.4 ± 1.0 | 10.2 ± 1.0 | 15.4 ± 1.9 |
| $A_{20}$ | 22.3 ± 3.1 | 17.3 ± 2.5 | 26.4 ± 6.9 |
| $^{211}$At-BSA | | | |
| $A_{50}$ | 15.0 ± 1.8 | 15.5 ± 0.9 | 26.8 ± 2.1 |
| $A_{20}$ | 32.7 ± 15.9 | 32.8 ± 3.1 | 48.3 ± 12.6 |

Added activity (± s.e.) in kBq ml$^{-1}$ in the culture medium to give 50% ($A_{50}$) and 20% ($A_{20}$) survival of microcolonies calculated using linear activity-log survival interpolation between experimental points (Figures 3 and 4). The $^{211}$At preparations were allowed to decay completely (> 72 h) before the medium was changed. s.e. values were calculated from the s.e. values of the two nearest experimental points.

**Table V** Therapeutic gain factors

| *Cell line* | *OHS/WIX-c* | *KPDX/WIX-c* |
|---|---|---|
| 24 h microcolonies | | |
| Free $^{211}$At | | |
| $TGF_{50}$ | $0.6 \pm 0.1$ | $0.7 \pm 0.1$ |
| $TGF_{20}$ | $0.7 \pm 0.3$ | $1.0 \pm 0.3$ |
| $^{211}$At-BSA | | |
| $TGF_{50}$ | $0.8 \pm 0.2$ | $0.8 \pm 0.1$ |
| $TGF_{20}$ | $0.9 \pm 0.3$ | $1.0 \pm 0.3$ |
| 24 h microcolonies | | |
| 14 MBq mg$^{-1}$ $^{211}$At-TP-3 | | |
| $TGF_{50}$ | $2.5 \pm 0.5$ | $2.3 \pm 0.3$ |
| $TGF_{20}$ | $2.0 \pm 0.6$ | $1.3 \pm 0.4$ |
| 50 MGq $^{-1}$ $^{211}$At-TP-3 | | |
| $TGF_{50}$ | $3.7 \pm 0.5$ | $1.8 \pm 0.2$ |
| $TGF_{20}$ | $3.8 \pm 1.0$ | $1.5 \pm 0.3$ |
| 72 h microcolonies | | |
| 50 MBq mg$^{-1}$ $^{211}$At-TP-3 | $2.9 \pm 0.6$ | $2.7 \pm 0.4$ |

The therapeutic gain factors for 50% ($TGF_{50}$) and 20% ($TGF_{20}$) microcolonies survival. TGF ($\pm$ s.e.) was calculated according to the following equation (e.g. $TGF_{50}$, OHS, $^{211}$At-BSA):

$TGF = [D_{50}$ (OHS, X-rays)$/A_{50}$ (OHS, $^{211}$At-BSA)]/[$D_{50}$ (WIX-c, X-rays)$/A_{50}$ (WIX-c, $^{211}$At-BSA)].

**Table VI** Therapeutic gain factors for $^{211}$At-TP-3 *vs* $^{211}$At-BSA

| *Cell line* | *OHS* | *KPDX* | *WIX-c* |
|---|---|---|---|
| 14 MBq mg$^{-1}$ | | | |
| $TGF_{50}$ | $2.9 \pm 0.6$ | $2.7 \pm 0.4$ | $0.9 \pm 0.1$ |
| $TGF_{20}$ | $2.1 \pm 0.6$ | $1.3 \pm 0.3$ | $1.0 \pm 0.3$ |
| 50 MBq mg$^{-1}$ | | | |
| $TGF_{50}$ | $4.5 \pm 0.7$ | $2.3 \pm 0.2$ | $1.0 \pm 0.1$ |
| $TGF_{20}$ | $4.2 \pm 1.0$ | $1.6 \pm 0.3$ | $1.0 \pm 0.3$ |

The therpeutic gain factors were determined for 50% ($TGF_{50}$) and 20% ($TGF_{20}$) survival of 24 h microcolonies. TGF ($\pm$ s.e.) was calculated according to the following equation (e.g. OHS):

$TGF = [A_{50}$ (OHS, $^{211}$At-BSA)$/A_{50}$ (OHS, $^{211}$At-TP-3)].

the short range of the α-particles (<80 μm) the radiation dose to the target cells mainly comes from cell-bound $^{211}$At-MAbs. The dose is therefore under ideal conditions (i.e. all target cells contain antigens to the MAb) proportionally dependent on the number of $^{211}$At bound to the cells. At antigen saturation, this number is proportional to the specific activity of the MAb. Optimisation of immunoreactivity, specific activity of $^{211}$At-MAb and clearance of non-bound $^{211}$At-MAb will therefore increase the therapeutic gain further.

The therapeutic gain was in this study measured in two ways. Firstly, TGF values were determined for the antigen-positive cell lines using the antigen-negative cell line as reference. In this case corrections for differences in X-ray sensitivity were included. Secondly, TGF values were determined by comparison of the cytotoxicity of the cell-binding $^{211}$At-MAb with that of a non-specific $^{211}$At-protein using the same cell line. The target tissue (OHS and KPDX osteosarcoma microcolonies) was more sensitive to X-rays than the control tissue (WIX-c melanoma microcolonies). A system with resistant tumour cells and sensitive control cells would have given higher therapeutic gain. This is because cell lines generally show less variability in radiation response when treated with high linear energy transfer (LET) radiation, e.g. α-particles and slow neutrons, than when treated with low-LET radiation (Hall, 1988).

A characteristic that may be important for the therapeutic gain of α-particle RIT is the growth pattern of the cells within a microcolony. Microscopical examinations revealed that the OHS cells tended to grow in a scattered pattern while the KPDX cells tended to grow tightly in the microcolonies. The α-particle intra-colony cross-fire was therefore more important for the KPDX than for the OHS microcolonies. This explains why the 72 h microcolonies had significantly higher TGF values than the 24 h microcolonies for KPDX but not for OHS.

The experiments with $^{211}$At-BSA and $^{211}$At-TP-3 on the antigen-free WIX-c cell line show that the change in molecular size from approximate molecular weight (MW) of 66,000 to approximate MW of 160,000 did not influence the cytotoxicity of the $^{211}$At-protein conjugate. Moreover a change in the specific activity of $^{211}$At-TP-3 from 14 to 50 MBq mg$^{-1}$ did not change the non-specific cytotoxicity against WIX-c microcolonies significantly. It can therefore be concluded that the cytotoxicity of non-specific $^{211}$At-labelled proteins is not significantly influenced by the size and the specific activity of the $^{211}$At-protein conjugate.

The uptake of radiolabelled MAbs measured in tumours in patients has generally been low (Epenetos *et al*., 1986). Clinically, intravenously injected short-lived α-particle-emitting radioimmunoconjugates such as $^{211}$At-TP-3 cannot be expected to give substantial therapeutic gain in the treatment of large solid tumours. The *in vitro* study presented here indicates, however, that a well-vascularised tumour or micrometastases may be candidates for treatment with $^{211}$At-MAbs, on the conditions that the MAb binds strongly to the tumour cells and has a low cross-reactivity with normal tissue. Further *in vivo* studies with relevant tumour models are therefore desirable.

The technical assistance of Eivind Olsen, Department of Physics, University of Oslo, during the cyclotron irradiations is gratefully acknowledged. Financial support was received from The Norwegian Cancer Society, Grant 90077.

### References

BLOOMER, W.D., MCLAUGHLIN, W.H., NEIRINCKX, R.D., ADELSTEIN, S.J., GORDON, P.R., RUTH, T.J. & WOLF, A.P. (1981). Astatine-211 cures experimental malignant ascites. *Science*, **212**, 340-341.

BLOOMER, W.D., MCLAUGHLIN, W.H., LAMBRECHT, R.M., ATCHER, R.W., MIRZADEH, S., MADARA, J.L., MILIUS, R.A., ZALUTSKY, M.R., ADELSTEIN, S.J. & WOLF, A.P. (1984). $^{211}$At radiocolloid therapy: further observations and comparison with radiocolloids of $^{32}$P, $^{165}$Dy, and $^{90}$Y. *Int. J. Radiat. Oncol. Biol. Phys.*, **10**, 341-348.

BROWN, G., BATEMAN, W., COWAN, J., FISHER, A.G., TOKSOZ, D. & VAUGHAN, A.T.M. (1981). Analyses of human haemopoietic precursor cell antigens using astatine labelled monoclonal antibodies. *Protides Biol. Fluids*, **29**, 937-940.

BROWN, I. (1986). Astatine-211: Its possible applications in cancer therapy. *Appl. Radiat. Isot.*, **37**, 789-798.

BRULAND, Ø., FODSTAD, Ø. & PIHL, A. (1985). The use of multicellular spheroids in establishing human sarcoma cell lines *in vitro*. *Int. J. Cancer*, **35**, 793-798.

BRULAND, Ø., FODSTAD, Ø., FUNDERUD, S. & PIHL, A. (1986). New monoclonal antibodies specific for human sarcomas. *Int. J. Cancer*, **38**, 27-31.

BRULAND, Ø., FODSTAD, Ø., STENWIG, A.E. & PIHL, A. (1988). Expression of a novel human osteosarcoma-associated cell surface antigen. *Cancer Res.*, **48**, 5302-5309.

EPENETOS, A.A., SNOOK, D., DURBIN, H., JOHNSON, P.M. & TAYLOR-PAPADIMITROU, J. (1986). Limitations of radiolabelled monoclonal antibodies for localisation of human neoplasms. *Cancer Res.*, **46**, 3183-3191.

FODSTAD, Ø., BRØGGER, A., BRULAND, Ø.S., SOLHEIM, Ø.P., NESLAND, J.M. & PIHL, A. (1986). Characteristics of a cell line established from a patient with multiple osteosarcoma, appearing 13 years after treatment for bilateral retinoblastoma. *Int. J. Cancer*, **38**, 33-40.

GARG, P.K., ARCHER, G.E., BIGNER, D.D. & ZALUTSKY, M.R. (1989). Synthesis of radioiodinated N-succinimidyl iodobenzoate: optimization for use in antibody labelling. *Appl. Radiat. Isot.*, **40**, 485-490.

HALL, E.J. (1988). *Radiobiology for the Radiologist*, 3rd edn. J.B. Lippincott: Philadelphia.

HARRISON, A. & ROYLE, L. (1987). Efficacy of astatine-211-labeled monoclonal antibody in treatment of murine T-cell lymphoma. *Natl Cancer Inst. Monogr.*, **3**, 157–158.

HUMM, J.L. (1987). A microdosimetric model of astatine-211 labelled antibodies for radioimmunotherapy. *Int. J. Radiat. Oncol. Biol. Phys.*, **13**, 1767–1773.

HUMM, J.L. & CHIN, L.M. (1993). A model of cell inactivation by alpha-particle internal emitters. *Radiat. Res.*, **134**, 143–150.

KOZAK, R.W., ATCHER, R.W., GANSOW, O.A., FRIEDMAN, A.M., HINES, J.J. & WALDMAN, T.A. (1986). Bismuth-212-labelled anti-Tac monoclonal antibody: α-particle-emitting radionuclides as modalities for radioimmunotherapy. *Proc. Natl Acad. Sci. USA*, **83**, 474–478.

KURTZMAN, S.H., RUSSO, A., MITCHELL, J.B., DEGRAFF, W., SINDELAR, W.F., BRECHBIEL, M.W., GANZOW, O.A., HINES, J.J., GAMESON, J. & ATCHER, R.W. (1988). $^{212}$Bismuth linked to an antipancreatic carcinoma antibody: model for alpha-particle-emitter radiotherapy. *J. Natl Cancer Inst.*, **80**, 449–452.

LARSEN, R.H., HASSFJELL, S.P., HOFF, P., ALSTAD, J., OLSEN, E., VERGOTE, I.B., DE VOS, L., BJØRGUM, J. & NUSTAD, K. (1993). $^{211}$At-labelling of polymer particles for radiotherapy: synthesis, purification and stability. *J. Labelled Compounds Radiopharm.*, **33**, 977–986.

LARSEN, R.H., BRULAND, Ø.S., HOFF, P., ALSTAD, J., LINDMO, T. & ROFSTAD, E.K. (1994). Inactivation of human osteosarcoma cells *in vitro* by $^{211}$At-TP-3 monoclonal antibody: comparison with $^{211}$At labelled bovine serum albumin, free $^{211}$At, and external beam X-rays. *Radiat. Res.* (in press).

LINDMO, T., BOVEN, E., CUTTITTA, F., FEDORKO, J. & BUNN, P.A. (1984). Determination of immunoreactive fraction of radiolabelled monoclonal antibodies by linear extrapolation to binding at infinite antigen excess. *J. Immun. Methods*, **72**, 77–89.

LINK, E.M., BROWN, I., CARPENTER, R.N. & MITCHELL, J.S. (1989). Uptake and therapeutic effectiveness of $^{125}$I-and $^{211}$At-methylene blue for pigmented melanoma in an animal model system. *Cancer Res.*, **49**, 4332–4337.

MACKLIS, R.M., KAPLAN, W.D., FERRARA, J.L., ATCHER, R.W., HINES, J.J., BURAKOFF, S.J. & COLEMAN, C.N. (1989). Resident assay award: alpha particle radio-immunotherapy: animal models and clinical prospects. *Int. J. Radiat. Oncol. Biol. Phys.*, **16**, 1377–1387.

ROFSTAD, E.K. (1992). Retention of cellular radiation sensitivity in cell and xenograft lines established from human melanoma surgical specimens. *Cancer Res.*, **52**, 1764–1769.

ROFSTAD, E.K., WAHL, A., HYSTAD, M.E., NESLAND, J.M. & STOKKE, T. (1991). Establishment in monolayer culture and characterization of four human melanoma cell lines. *Virchows Arch. B Cell Pathol.*, **60**, 189–197.

VAUGHAN, A.T.M., BATEMAN, W.J. & COWAN, J. (1981). The preparation and cytotoxic properties of $^{211}$At-labelled concanavalin A bound to cell membranes. *J. Radioanal. Chem.*, **64**, 33–39.

VAUGHAN, A.T.M., BATEMAN, W.J., BROWN, G. & COWAN, J. (1982). The specific inhibition of cellular clonogenic proliferation using $^{211}$At labelled lectins and antibodies-I. *Int. J. Nucl. Med. Biol.*, **9**, 167–171.

VERGOTE, I.B., LARSEN, R.H. DE VOS, L.N., NESLAND, J.M., BRULAND, Ø.S., BJØRGUM, J., ALSTAD, J., TROPÉ, C.G. & NUSTAD, K. (1992). Therapeutic efficacy of the α-emitter $^{211}$At bound on microspheres compared with $^{90}$Y and $^{32}$P colloids in a murine intraperitoneal tumor model. *Gynecol. Oncol.*, **47**, 366–372.

ZALUTSKY, M.R. & NARULA, A.S. (1987). A method for the radiohalogenation of proteins resulting in decreased thyroid uptake of radioiodine. *Appl. Radiat. Isot.*, **38**, 1051–1055.

ZALUTSKY, M.R., GARG, P.K., FRIEDMAN, H.S. & BIGNER, D.D. (1989). Labeling monoclonal antibodies and F(ab′)$_2$ fragments with the α-particle-emitting nuclide astatine-211: preservation of immunoreactivity and *in vivo* localizing capacity. *Proc. Natl Acad. Sci. USA*, **86**, 7149–7153.


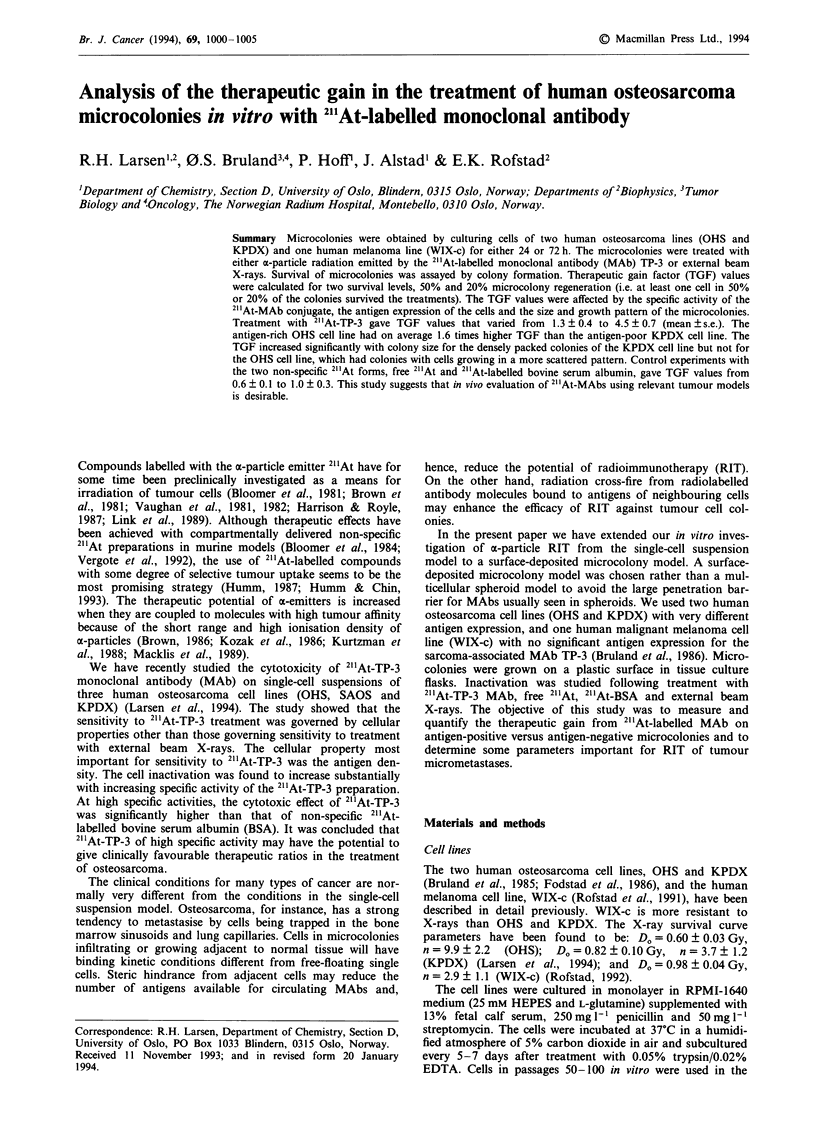

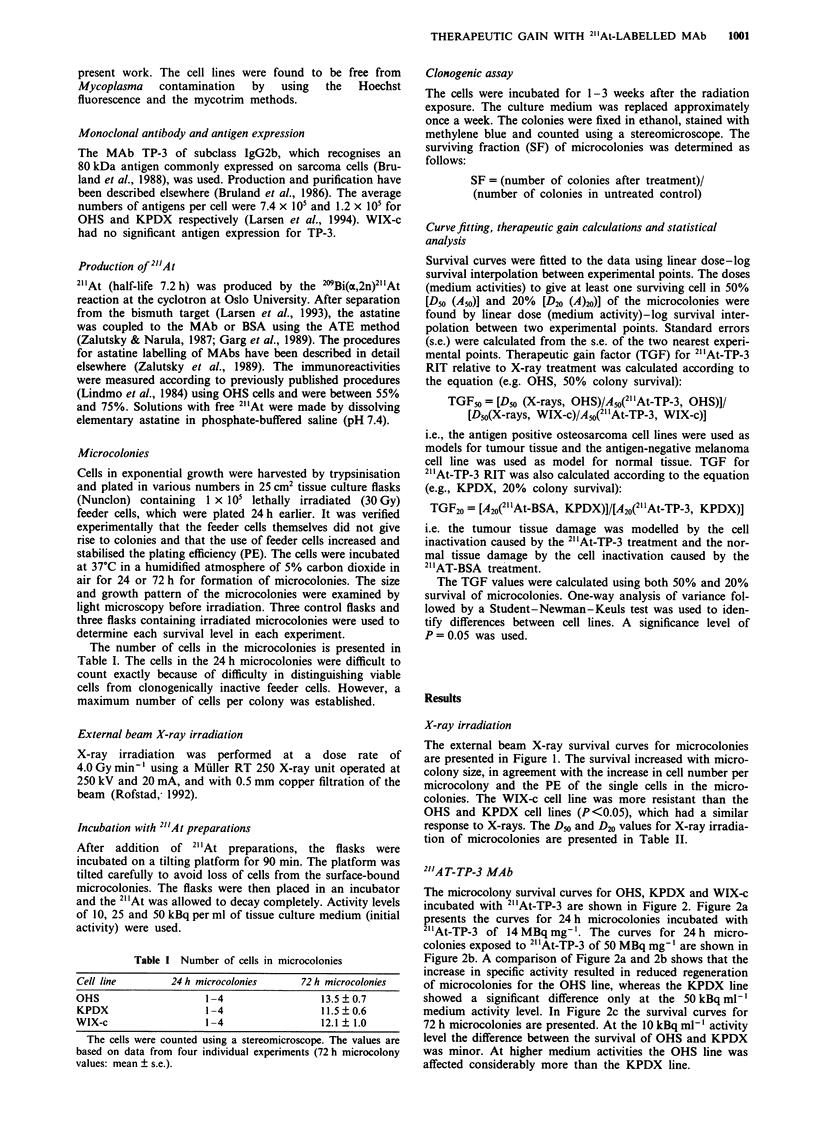

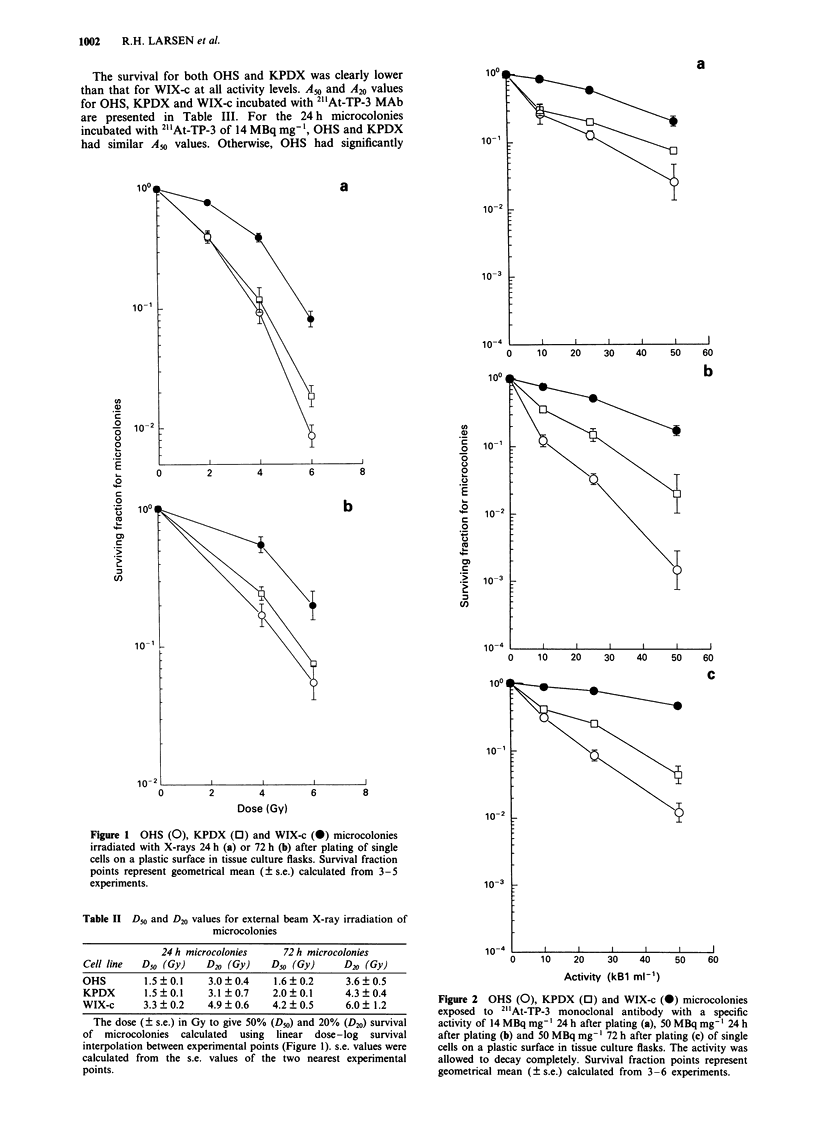

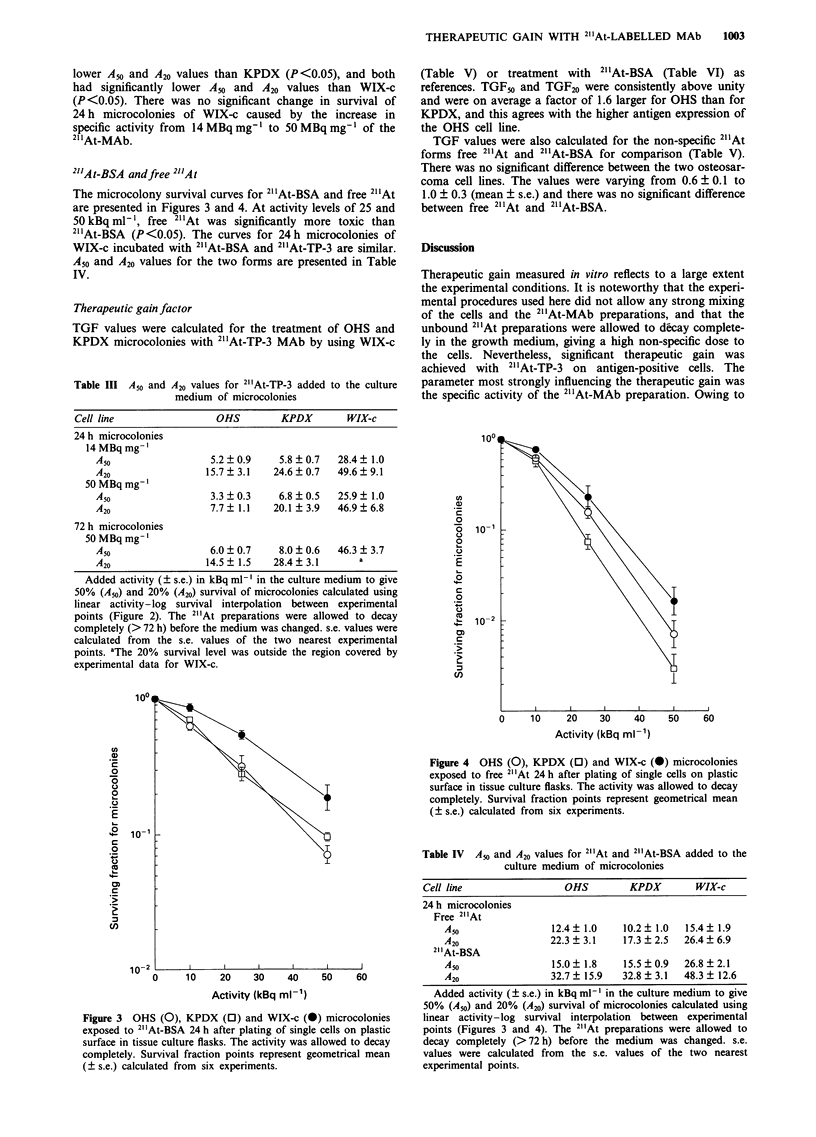

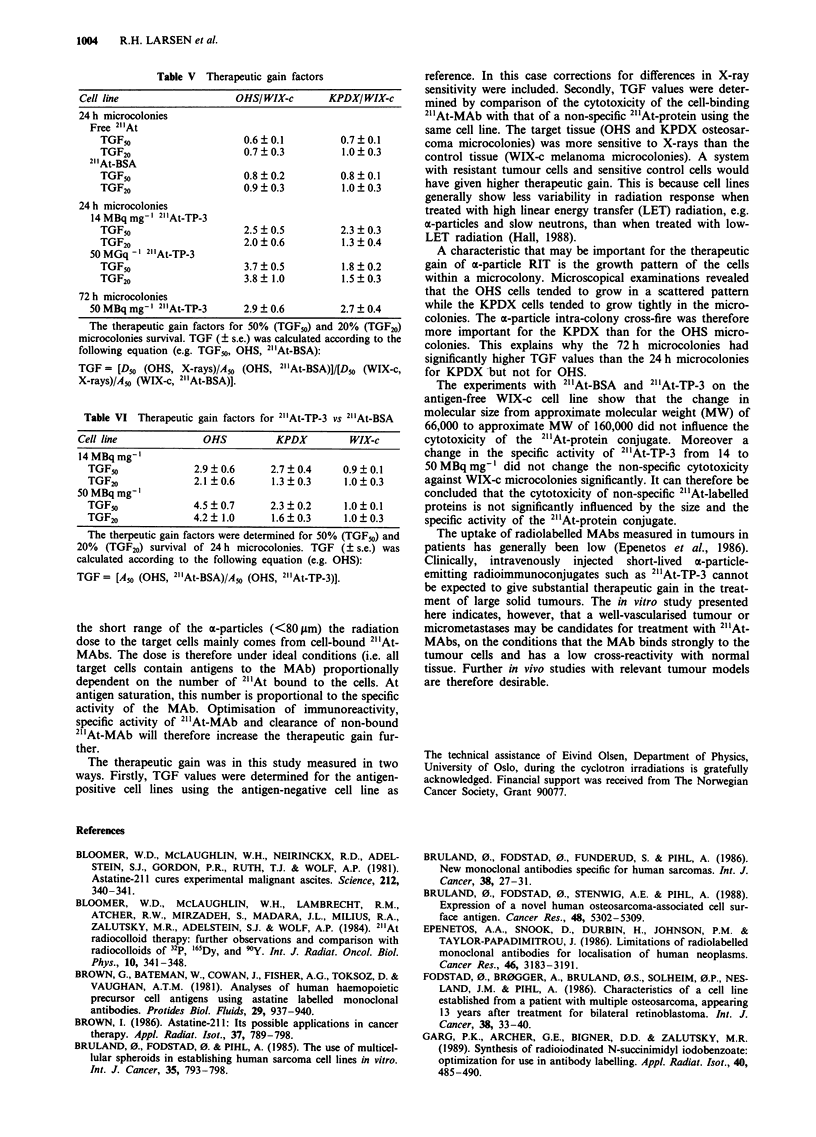

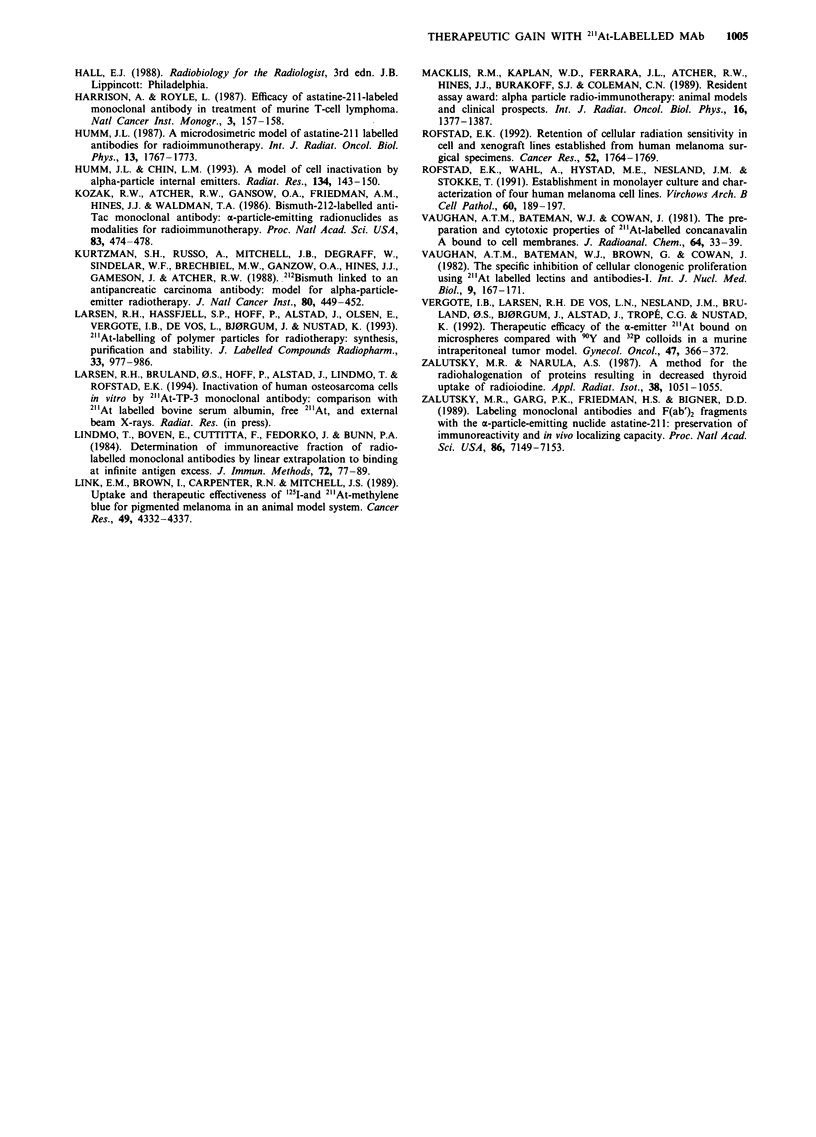


## References

[OCR_00741] Bloomer W. D., McLaughlin W. H., Lambrecht R. M., Atcher R. W., Mirzadeh S., Madara J. L., Milius R. A., Zalutsky M. R., Adelstein S. J., Wolf A. P. (1984). 211At radiocolloid therapy: further observations and comparison with radiocolloids of 32P, 165Dy, and 90Y.. Int J Radiat Oncol Biol Phys.

[OCR_00737] Bloomer W. D., McLaughlin W. H., Neirinckx R. D., Adelstein S. J., Gordon P. R., Ruth T. J., Wolf A. P. (1981). Astatine-211--tellurium radiocolloid cures experimental malignant ascites.. Science.

[OCR_00755] Brown I. (1986). Astatine-211: its possible applications in cancer therapy.. Int J Rad Appl Instrum A.

[OCR_00769] Bruland O. S., Fodstad O., Stenwig A. E., Pihl A. (1988). Expression and characteristics of a novel human osteosarcoma-associated cell surface antigen.. Cancer Res.

[OCR_00764] Bruland O., Fodstad O., Funderud S., Pihl A. (1986). New monoclonal antibodies specific for human sarcomas.. Int J Cancer.

[OCR_00759] Bruland O., Fodstad O., Pihl A. (1985). The use of multicellular spheroids in establishing human sarcoma cell lines in vitro.. Int J Cancer.

[OCR_00774] Epenetos A. A., Snook D., Durbin H., Johnson P. M., Taylor-Papadimitriou J. (1986). Limitations of radiolabeled monoclonal antibodies for localization of human neoplasms.. Cancer Res.

[OCR_00782] Fodstad O., Brøgger A., Bruland O., Solheim O. P., Nesland J. M., Pihl A. (1986). Characteristics of a cell line established from a patient with multiple osteosarcoma, appearing 13 years after treatment for bilateral retinoblastoma.. Int J Cancer.

[OCR_00787] Garg P. K., Archer G. E., Bigner D. D., Zalutsky M. R. (1989). Synthesis of radioiodinated N-succinimidyl iodobenzoate: optimization for use in antibody labelling.. Int J Rad Appl Instrum A.

[OCR_00799] Harrison A., Royle L. (1987). Efficacy of astatine-211-labeled monoclonal antibody in treatment of murine T-cell lymphoma.. NCI Monogr.

[OCR_00804] Humm J. L. (1987). A microdosimetric model of astatine-211 labeled antibodies for radioimmunotherapy.. Int J Radiat Oncol Biol Phys.

[OCR_00809] Humm J. L., Chin L. M. (1993). A model of cell inactivation by alpha-particle internal emitters.. Radiat Res.

[OCR_00813] Kozak R. W., Atcher R. W., Gansow O. A., Friedman A. M., Hines J. J., Waldmann T. A. (1986). Bismuth-212-labeled anti-Tac monoclonal antibody: alpha-particle-emitting radionuclides as modalities for radioimmunotherapy.. Proc Natl Acad Sci U S A.

[OCR_00820] Kurtzman S. H., Russo A., Mitchell J. B., DeGraff W., Sindelar W. F., Brechbiel M. W., Gansow O. A., Friedman A. M., Hines J. J., Gamson J. (1988). 212Bismuth linked to an antipancreatic carcinoma antibody: model for alpha-particle-emitter radioimmunotherapy.. J Natl Cancer Inst.

[OCR_00841] Lindmo T., Boven E., Cuttitta F., Fedorko J., Bunn P. A. (1984). Determination of the immunoreactive fraction of radiolabeled monoclonal antibodies by linear extrapolation to binding at infinite antigen excess.. J Immunol Methods.

[OCR_00847] Link E. M., Brown I., Carpenter R. N., Mitchell J. S. (1989). Uptake and therapeutic effectiveness of 125I- and 211At-methylene blue for pigmented melanoma in an animal model system.. Cancer Res.

[OCR_00853] Macklis R. M., Kaplan W. D., Ferrara J. L., Atcher R. W., Hines J. J., Burakoff S. J., Coleman C. N. (1989). Alpha particle radio-immunotherapy: animal models and clinical prospects.. Int J Radiat Oncol Biol Phys.

[OCR_00860] Rofstad E. K. (1992). Retention of cellular radiation sensitivity in cell and xenograft lines established from human melanoma surgical specimens.. Cancer Res.

[OCR_00865] Rofstad E. K., Wahl A., Hystad M. E., Nesland J. M., Stokke T. (1991). Establishment in monolayer culture and characterization of four human melanoma cell lines.. Virchows Arch B Cell Pathol Incl Mol Pathol.

[OCR_00876] Vaughan A. T., Bateman W. J., Brown G., Cowan J. (1982). The specific inhibition of cellular clonogenic proliferation using 211At labelled lectins and antibodies--I.. Int J Nucl Med Biol.

[OCR_00884] Vergote I., Larsen R. H., de Vos L., Nesland J. M., Bruland O., Bjørgum J., Alstad J., Tropé C., Nustad K. (1992). Therapeutic efficacy of the alpha-emitter 211At bound on microspheres compared with 90Y and 32P colloids in a murine intraperitoneal tumor model.. Gynecol Oncol.

[OCR_00894] Zalutsky M. R., Garg P. K., Friedman H. S., Bigner D. D. (1989). Labeling monoclonal antibodies and F(ab')2 fragments with the alpha-particle-emitting nuclide astatine-211: preservation of immunoreactivity and in vivo localizing capacity.. Proc Natl Acad Sci U S A.

[OCR_00889] Zalutsky M. R., Narula A. S. (1987). A method for the radiohalogenation of proteins resulting in decreased thyroid uptake of radioiodine.. Int J Rad Appl Instrum A.

